# Differential Measurements of Oxidatively Modified Proteins in Colorectal Adenopolyps

**DOI:** 10.4236/ijcm.2015.64037

**Published:** 2015-04-27

**Authors:** Sharifeh Mehrabi, Lashanale Wallace, Shakeria Cohen, Xuebiao Yao, Felix O. Aikhionbare

**Affiliations:** 1 Department of Medicine, Morehouse School of Medicine, Atlanta, USA; 2 Department of Physiology, Morehouse School of Medicine, Atlanta, USA

**Keywords:** Colorectal Adenopolyps, Oxidative Stress, Carbonyl Proteins

## Abstract

**Introduction:**

Adenopolyps patients have a three-fold higher risk of colon cancer over the general population, which increases to six-fold if the polyps are multiple and with lower survival among African American population. Currently, 6% of CRC can be ascribed to mutations in particular genes. Moreover, the optimal management of patients with colorectal adenopolyps depends on the accuracy of appropriate staging strategies because patients with similar colorectal adenocarcinoma architecture display heterogeneity in the course and outcome of the disease. Oxidative stress, due to an imbalance between reactive oxygen species (ROS) and antioxidant capacities as well as a disruption of redox signaling, causes a wide range of damage to DNA, proteins, and lipids which promote tumor formation.

**Objective/Method:**

This study applied spectrophotometric, dinitrophenylhydrazone (DNPH) assay, two-dimensional gel electrophoresis, and western blot analyses to assess the levels of oxidatively modified proteins in 41 pairs of primary colorectal tissues including normal/surrounding, adenopolyps (tubular, tubulovillous, villous, polypvillous) and carcinoma. Analysis of variance (ANOVA) and Student’s *t*-tests were utilized for the resulting data set.

**Results:**

Our data showed that the levels of reactive protein carbonyl groups significantly increased as colorectal adenopolyps progresses to malignancy. No significant differences were found in the levels of carbonyl proteins between gender samples analyzed. For African American patients, there were, relative to Caucasians, 10% higher levels of reactive carbonyls in proteins of tubulovillous tissue samples (*P* < 0.05) and over 36% higher in levels in adenocarcinomas (*P* < 0.05). In normal tissues and tubular, there were no significant differences between the two groups in levels of protein carbonyls. Differences in the levels of protein carbonyl expression within individual patient samples with different number of tumor cells were notably evident.

**Conclusion:**

Results suggested that oxidative stress could be involved in the modification of oxidatively carbonyl proteins in the precancer stages, leading to increased aggressiveness of colorectal polyps.

## 1. Introduction

Despite the recent advances achieved in early diagnosis and treatment, colorectal cancer (CRC) is one of the main causes of cancer related deaths worldwide [1]. Previous study by Fearon and Vogelstein [2], established that the appearance of CRC from normal colonic mucosa to carcinoma was mediated by mutations in the genes that control the cell cycle (proliferation, differentiation, adhesion, and apoptosis) or in the DNA repair genes. The colonic cell types are transformed from tubular adenoma, tubulovillous, and villous components to carcinoma. However, the initial event responsible for transformation of normal cells of the colonic mucosa into neoplastic cells of tubular, tubulovillous and villous components still has not been fully clarified.

Generally, oxidative stress promotes damage to cellular proteins, lipids, membranes and DNA, and plays a key role in the development of cancer. ROS disrupt redox homeostasis and promote tumor formation by initiating aberrant activation of signaling pathways that lead to tumorigenesis [3]. Colonic cells are constantly exposed to increased formation of ROS in the intestinal lumen and continuous exposure of the mucosa to these free radicals resulting from dietary consumption promote oxidative damage to the DNA of the epithelial cells, thereby triggering the appearance of genetic mutations [4]. When these mutations harm the genes responsible for controlling the cell cycle or the DNA repair system, cell clones with proliferative autonomy can emerge, thus representing the initial mechanism for carcinogenesis.

The chronic aggressive action on the colonic mucosa caused by oxygen-reactive species gives rise to a chronic inflammatory process that progressively modifies the normal architecture of the colonic epithelium which promotes the appearance of areas with increasing degrees of tissue dysplasia [5]. The close relationship that exists between chronic intestinal inflammatory diseases (such as in ulcerative colitis and Crohn’s disease) and CRC seems to reinforce this evidence [6]-[8]. The more intense and long-lasting oxidative aggression on the mucous epithelium of the colon is, the greater the risk of neoplasia in form of adenopolyps.

The risk of developing adenopolyps type of CRC increases with advancing age and the levels of oxidative modified proteins, carbonyl contents that are usually used to measure ROS in cells, have been implicated to increase in a variety of disease processes, notably during aging. Given that the electron transport chains in the mitochondria and endoplasmic reticulum are most important sources of ROS in pre-neoplastic and cancer cells, they are metabolically active and require high levels of ATP to maintain their higher-than-normal proliferation rates [9]. In this study, we evaluate protein carbonyl content levels in colorectal adenopolyps tissue samples, including samples obtained from African Americans and Caucasians, in order to determine the role of oxidative stress during colorectal tumoriogenesis. We also assess the relationship of carbonyl content levels among individual patients with the disease.

## 2. Materials and Methods

### 2.1. Study Samples

Forty one pairs of primary colorectal tumor tissues that were from the Southern Regional Cooperative Human Tissue Network which were previously obtained from patients that underwent surgical resections and 41 of these samples were colorectal adenopolyps, comprising of tubular, tubulovillous, villous, polypvillous and carcinomas, 41 of their normal/surrounding tissues. Of these 41 pairs tissue samples, 11 pairs were from African Americans and 27 pairs were from Caucasians and 3 pairs of the samples were demographic unknown. Seventeen pairs of the samples were obtained from men and 21 pairs of female with mean age of 55 ± 10 years (**Table 1**).

All tissue samples were micro-dissected, diagnosed and histopathologically confirmed by pathologists. Tumor stages were determined on the basis of criteria outlined by the American Joint Committee on Cancer (AJCC) and the demographic characteristics of the patients were grouped based on the clinical diagnosis as displayed in **Table 1**. These samples were stabilized by snap-freezing immediately after excision and dissection. The dissected tissues were placed in cryovials and immersed in liquid nitrogen. All samples were transferred to −80°C for long-term storage as recommended for measurement of proteins with reactive carbonyl groups [10].

All studies were implemented under protocols approved by Institutional Review Boards of Morehouse School of Medicine and the University of Alabama at Birmingham.

### 2.2. Extraction of Cytosolic Fractions

The cytosolic fractions of the tissue samples were prepared by differential centrifugation using mitochondria/cytosol fractionation kits (BioVision, CA). Approximately 400 mg of each sample was cut into small pieces and placed in a 2 ml plastic tube on ice and washed twice with ice-cold phosphate-buffered saline (PBS). Each tissue sample was mildly homogenized in an ice-cold dounce tissue grinder and centrifuged at 700 × g for 5 min at 4°C. The supernatant was removed, and 1 ml of homogenizing buffer containing protease inhibitors was added. The sample was incubated on ice for 10 min and homogenized in an ice-cold Dounce tissue grinder, with about 50 - 60 passes. The homogenate was then transferred into a 1.5 ml microcentrifuge tube and centrifuged at 700 × g for 10 min at 4°C. The supernatant was collected, transferred to a fresh 1.5 ml microcentrifuge tube, and centrifuged at 10,000 × g for 30 min at 4°C. The supernatant, the cytosolic fraction, was collected. This fraction was treated with a 1% streptomycin sulfate solution for 15 min to remove DNA, which could react with DNPH and contribute to the reactive carbonyl level of homogenates. After incubation, samples were centrifuged at 13,000 × g for 15 min at room temperature. The supernatant, a DNA-free preparation was removed and saved for DNPH assay.

### 2.3. Measurement of Total Protein Concentration

A microplate DC protein assay (BioRad) was used to measure the protein contents of the samples. Each sample was analyzed in duplicate, and a pooled tissue sample was included in each plate to estimate the inter-assay coefficient of variation. A fraction of 5 µl of each sample and standard protein (BSA) was added to the well of a 96 wells microplate followed by adding 200 µl of reagent A and B as recommended by BioRad protocol. The plate was placed on the plate mixer and mixed for 5 sec, and then incubated at room temperature for 15 min. The absorbance at 750 nm was determined spectrophotometrically, the protein concentration of each homogenate was extrapolated from a standard curve. Samples of an extract of MCF7 cells, and a protein extract from a control cell line were included in each run as positive controls.

### 2.4. Protein Carbonyl Assay

To assess patterns of oxidation, oxidized protein modifications in colorectal tumor samples were determined by measuring reactive protein carbonyl groups. ROS react with amino acid residues in protein, particularly histidine, arginine, lysine and proline, to produce carbonyl functions that can react with DNPH, leading to formation of stable dinitrophenylhydrazone adducts [11] [12]. This reaction is used to estimate reactive carbonyl content of proteins in human tissues and body fluid [10]. The protein carbonyl content of the homogenates was determined accordingly to Sigma kits. Briefly, homogenates of colorectal tumor tissue samples (110 µl) were placed in each mirocentrifuge tubes labeled as treated samples and untreated control respectively. To prevent nucleic acid interference 15 µl of 10% streptozocin solution (Sigma) was added to each sample. Samples were next incubated at room temperature for 15 minutes subsequently centrifuged according to Sigma’s protocol. A 100 µl of the supernatant was transferred to a fresh tube and one hundred µl of DNPH (Sigma) was added to each sample tube and incubated at room temperature for 10 minutes. The hydrazone derivatives were precipitated with 100% (wt/vol) trichloroacetic acid, and washed with 500 µl of acetone to remove excess DNPH. The pellet was dissolved in 6 M guanidine hydrochloride. Differences between optical densities of DNPH treated and water used as control samples were determined spectrophotometrically at 375 nm. The results were calculated as nmol of DNPH incorporated per mg of protein, as determined from absorptivity using the Bear-Lambert equation and an extinction coefficient 22,000 µM^−1^·cm^−1^. To determine the stability of the samples, protein carbonyls were measured in a pooled tissue sample that was repeatedly frozen and thawed and there was no significant difference in protein [13] [14].

### 2.5. SDS-PAGE and Western Blot Analyses

In this study, to determine the number and relative mass of the DNPH derivatized proteins in the tissue samples, SDS-PAGE and Western blot immunoassays were performed using OxyBlot Protein Oxidation Detection Kits, (Millipore). Samples of DNPH-derivatized proteins were resolved on 10% SDS-polyacrylamide gels. As a control an underivatized sample of each cytosolic fraction was run along with DNPH-derivatized samples. The proteins were transferred to PVDF membranes blotted with rabbit anti-DNPH antibody and detected with a super-Singnal^®^ West Pico chemiluminescent substrate. A DNPH-derivatized standard protein was used for estimation of molecular weight. Proteins that were oxidatively modified were identified by their appearance as bands in the lane containing the derivatized sample, but not in the lane containing the control. Blots were quantified using the relative optical density on the level of reactive carbonyl proteins using myImageAnalysis v 1.1, Thermo Scientist.

### 2.6. Statistics

Data were analyzed using analysis of variance (ANOVA) and Student’s *t*-tests.

## 3. Results

To compare the extent of oxidative modification among normal, colorectal adenopolyps and carcinoma tissues, measurement of the reactive carbonyl groups as relates to the intensity of the oxidative stress is essential for better understanding of the etiopathogenic mechanisms of carcinogensis. Free radicals in biological systems generally lead to oxidative, post-translational modifications of proteins [15]. Proteins from lysates of samples of normal tissues and adenopolyps and invasive colorectal carcinomas were derivatized with DNPH to measure the levels of carbonyl groups by a spectrophotometric method.

**Figure 1:**
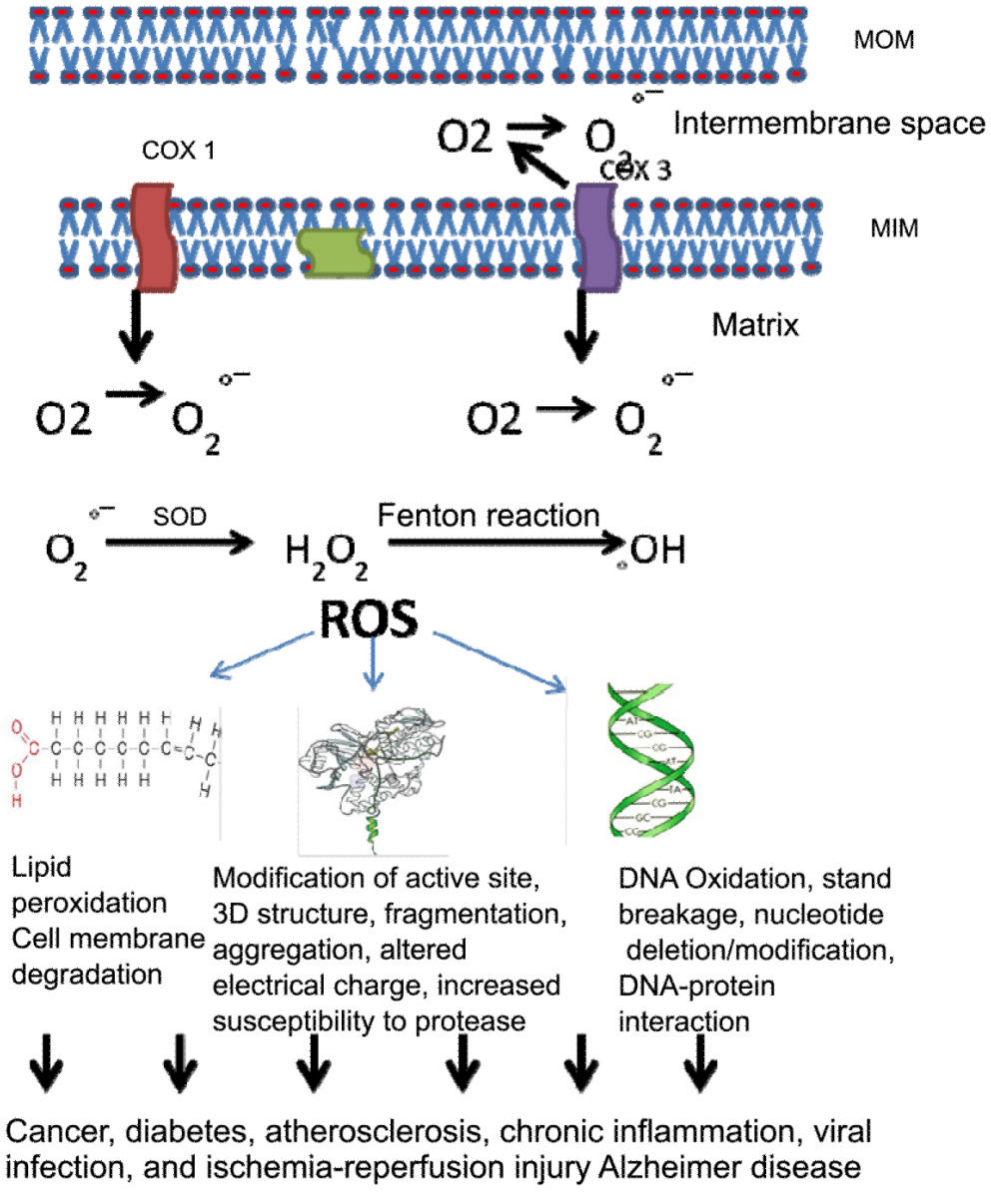
ROS production and its adverse effect on macromolecules. MOM: mitochondria outer, MIM: mitochondria inner membrane, ROS: reactive oxygen species, COXI: cytochrome oxydase I, SOD: superoxide dismutase.

**Figure 1** displays schematic diagraph of ROS production and its adverse effect on macromolecules. MOM; mitochondria outer, MIM; mitochondria inner membrane, ROS; Reactive Oxygen Species, COXI; cytochrome oxydase I, SOD; superoxide dismutase.

**Figure 2:**
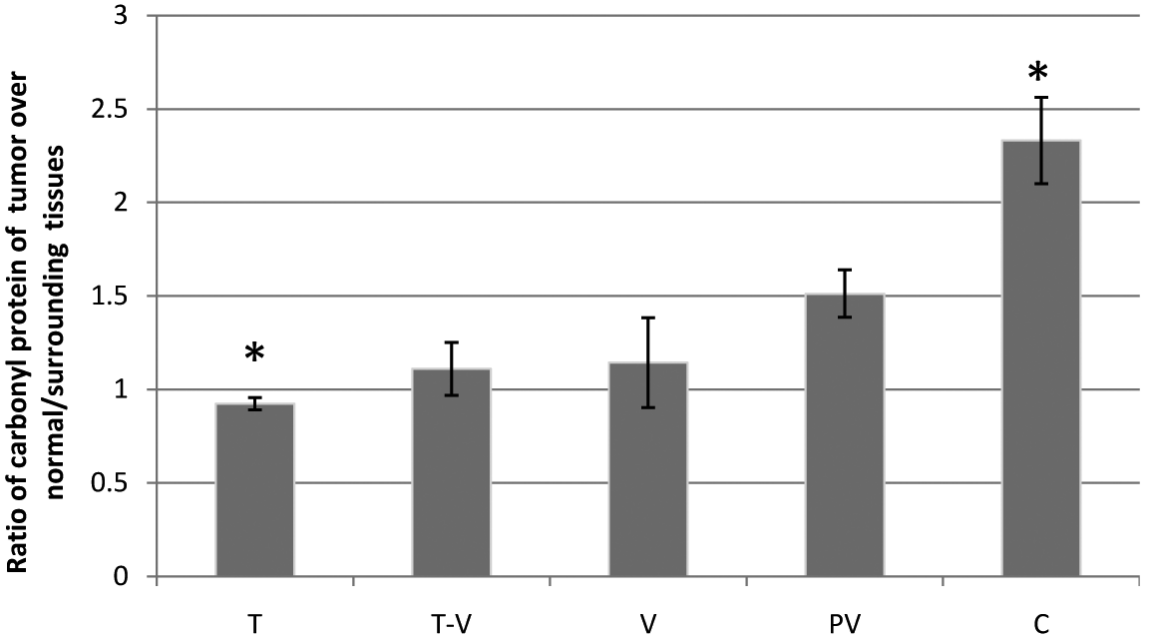
Levels of reactive carbonyl proteins in normal/surrounding colorectal adenopolyps and carcinoma tissues, as measured by the spectrophotometric method. T: tubular; T-V: tubulovillous; V: villous; PV: polypvillous, C: carcinoma. Significant differences between groups (*P* < 0.05)^*^, (n = 41 pairs samples).

**Figure 2** displays the data of the progressively increasing levels of carbonyl groups were observed in the derivatized lysates samples in the degree of disease advancement from tubular, tubulovillous, villous, polypvillous to adenocarcinomas.

**Figure 3:**
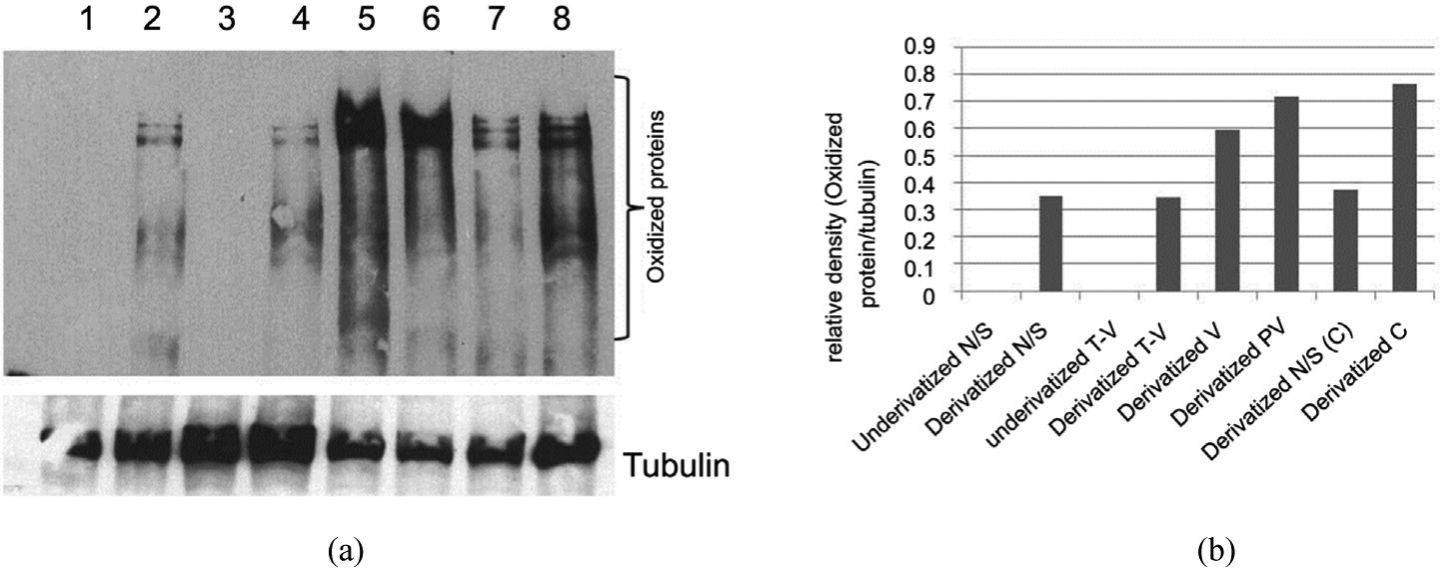
(a) Western blot analysis of oxidized protein in cytosolic fraction of colorectal adenopolyp tissue. Lane 1, underivatized lysate of normal tissue; Lane 2, DNPH-derivatized lysate of normal tissue; Lane 3, underivatized lysate of tubular villous tissue; Lane 4, DNPH-derivatized lysate of tubular villous tissue; Lane 5, DNPH-derivatized lysate of villous tissue; Lane 6, DNPH-derivatized lysate of polypvillous tissue; Lane 7, DNPH-derivatized lysate of normal tissue surrounding carcinoma tumor; Lane 8, DNPH-derivatized lysate of carcinoma tissue; (b) The relative optical density on the level of reactive carbonyl proteins using myImageAnalysis v 1.1, Thermo Scientist.

**Figure 3(a) and Figure 3(b)** display results from Western blots that showed a distinct difference in protein expression patterns between normal/surrounding, tubulovillous, villous and adenocarnoma tissues which were similar to those obtained with the spectrophotometric techniques. Lanes 1 and 3 are representatives of samples which were not DNPH-derivatized and used as negative controls for oxidized protein detection. DNPH are used as a marker for detection of oxidized protein and secondary antibody only detects the DNPH derivatized oxidized proteins. Relative to normal surrounding tissues, protein reactive carbonyls were elevated in adenopolyps tissues and notably, in the invasive stages and there were no significant differences were found in the levels of carbonyl proteins between gender samples analyzed.

**Figure 4:**
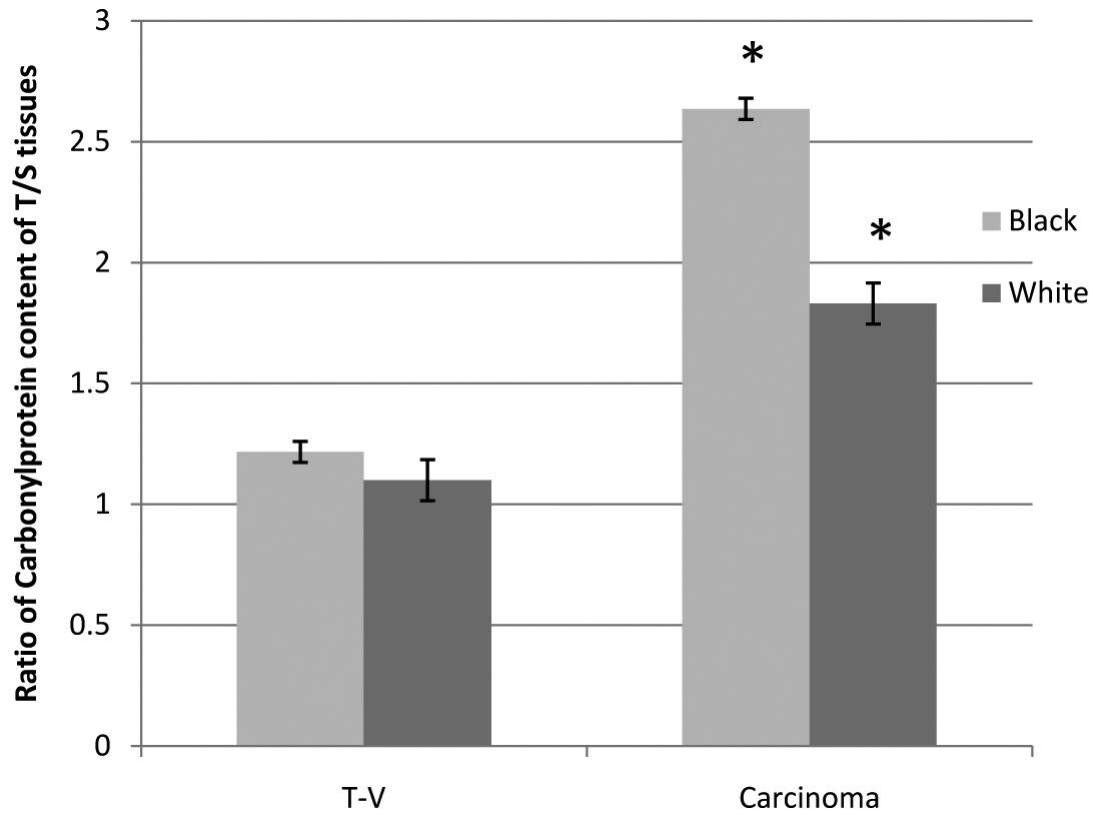
Levels of reactive carbonyl proteins in colorectal adenopolyps of tubular, tubulovillous, villous (T-V) and carcinoma as measured by the spectrophotometric method. There were significant differences between samples from African Americans (n = 11) and Caucasians (n = 27) (*P* < 0.05).

**Figure 4** displays data on the different levels of reactive carbonyl group between tissue samples from African Americans and Caucasians. For African Americans, there were, relative to Caucasians, 10% higher levels of reactive carbonyls in proteins of tubulovillous tissue samples (*P* < 0.05) and over 36% higher in levels in adenocarcinomas (*P* < 0.05). In normal tissues and tubular, there were no significant differences between the two groups in levels of protein carbonyls.

**Figure 5:**
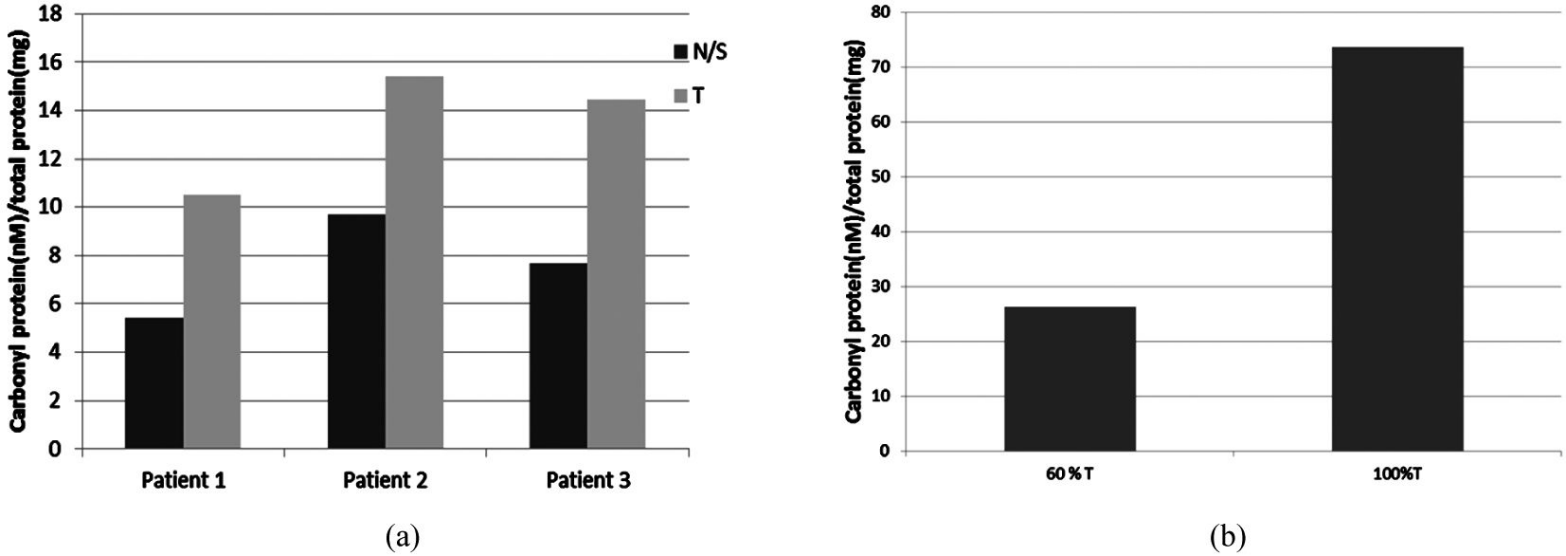
Levels of reactive carbonyl proteins in tumor tissue obtained from different patients as measured by the spectro-photometric method. (a) Levels of carbonyl proteins in normal/surrounding tissues relative to tumor tissues of the same patient; (b) Levels of carbonyl proteins in tissues with different degrees of tumor cells in an individual. N/S: normal/surrounding tissue; T: tumor tissue.

**Figure 5(a)** displays data that showed a 57%, 40%, and 54% higher levels of carbonyl protein in the tumors as compare to their normal surrounding tissues within respective three patients.

**Figure 5(b)** displays data that indicated tissue samples with higher contents of tumor cells exhibited higher levels of oxidized proteins relative to normal surrounding tissues and to samples with fewer tumor cells from same patient; A 70% of higher levels of oxidized proteins was observed within individual patient samples with high degree of tumor cells relative to normal surrounding tissues as compare to samples with fewer degree of tumor cells.

## 4. Discussion

The risk of carcinogenesis and other age-related diseases has been associated with oxidized proteins (protein carbonyls) [11] [16]-[20]. Both altered metabolism and inadequate tumor neovascularization may lead to an accumulation of ROS, by-products of oxidative phosphorylation. Recent studies have showed oxidative protein damage in carbonyl content in already transformed colorectal cancer tissues [3] [5] [21]. Since colorectal adenopolyps are derived from surface cell types of the colon and rectal epithelium oxidative modification may be implicated in the transformation of colorectal cancer tissues. Studies have shown that an imbalance of ROS can damage cellular proteins, lipid, carbohydrates and nucleic acids, which may be responsible for initiating and developing certain cancers–including colorectal cancer [22]. ROS protein damage is particularly significant due to its regulatory functions which could lead to ruptured polypeptide chains, cross link formation in or out of the same chain, and changes in structure of amino acids and complex proteins [23]. Rupture on the colonic mucosa involves tissue remodeling, with high cell turn-over, characteristic of inflammatory reactions, resulting from stimuli, such as cytokines (tumor necrosis factor and interleukin 1) and, bacterial toxins (lipopolysaccharide) [24]. Damage to colon epithelium resulting from inflammatory responses during metabolism is generally viewed as a secondary event. The primary event is the inflammatory cascade of neutrophil adherence to cell vasculature, disruption of their barrier, and subsequent infiltration of inflammatory cells into the interstitial space, leading to the release of oxidants and proteases resulting in luminal colon mucosal injury. A variety of chronic inflammatory conditions predispose susceptible cells to neoplastic transformation [25] and these inflammatory cytokines are such as TNF-*α* and ROS activate nuclear factor kappa-B (NF*κ*B). In its normal state, NF*κ*B is inhibited by its inhibitory protein (I*κ*B*α*), which downregulates the inflammatory response. In nuclei, NF*κ*B induces the expression of genes involved in cell proliferation, apoptosis, and carcinogenesis [26] and also induces production of proinflammatory cytokines, which enhance the inflammatory responses.

Effectors in the inflammatory response are ROS and these may directly or indirectly cause damage through their reactions with components of target cells [27]. ROS can also recruit other inflammatory cells, leading to additional ROS production and damage [28]. The most commonly used marker of protein oxidation is protein reactive carbonyl content [19] [20]. In cells oxidized proteins accumulate during aging, accompanied with oxidative stress and some pathological conditions [11] [19] [29] [30]. Furthermore, proteins modified by oxidative stress are associated with an increased risk of cancer [19] [31]. Recent studies have shown that there is enhanced oxidative stress in transformed colorectal cancer tissues relative to normal intestinal tissues [21] [32]. Similar results were found in this study in that it shows progressive elevated expression of protein carbonyls in adenopolyps as relative to normal surrounding tissues. These results suggest the progression of colorectal adenopolyps is associated with oxidative modification of proteins. Murilkewicz *et al.* [21] previously suggested that the reactions of ROS with proteins leads to modification of amino acids, modification of prosthetic groups, and aggregation or fragmentation of protein molecules with the progressive stages of colorectal tumor as observed in this study. Also, oxidative damaged proteins are not removed by proteosomic systems in an efficient manner, leading to its impaired function due to the accumulation in colorectal adenopolyps cells as observed in our results. Such accumulation of oxidized protein byproducts may be involved in the histopathogenesis of colorectal tumor similar to our previous study with serous ovarian tumor subtype [13]. Carcinogenesis in general may be mediated by oxidative damage to DNA, due to mutations in critical genes, such as the tumor suppressor *p*^53^ [33]. Damage to the *p*^53^ gene may reduce the effectiveness of DNA repair mechanisms, and increase the rate of cell division. Cells that are rapidly dividing cells are more prone to errors in DNA replication and repair [34] and may also be more sensitive to oxidative stress, enhancing the risk of carcinogenesis.

As demonstrated in this study, there are high levels of reactive protein carbonyls in tissue samples of colorectal adenopolyps and carcinomas from African Americans relative to Caucasians. This may be a result of racial differences in the intracellular levels of oxidized proteins, reflecting the imbalance between the rate of protein oxidation and the rate of oxidized protein degradation [35]. This imbalance could be a function of factors leading to the generation of ROS, such as dietary habits, alcohol consumptions and environmental processes which may lead to the formation of ROS [36]. Also, the imbalance could be as well as become factors in determining the concentrations and/or activities of the proteases that degrade oxidative damaged proteins [19]. Degradation is also dependent upon cellular components such as metal ions, inhibitors, activators, and regulatory proteins, which affect their proteolytic activities. For example, oxidized forms of some proteins, e.g., cross-linked proteins [37]-[39] and proteins modified by glycation [40] or by lipid peroxidation products [41], are resistant to proteolysis, and could lead to production of protease inhibitors that hinder degradation of the oxidized forms [37] [41]. Therefore, inactivation of these protein inhibitors could enhance the action of proteases, such as elastase, plasminogen activator, and plasmin. This process could facilitate tumoriogenesis, invasion and metastasis [42], particularly in various individuals or subgroups.

Past study has shown that African Americans with reduced dietary intake of antioxidants and impaired mitochondrial function may make them vulnerable to diseases associated with oxidative stress [43]. Basis of this statement is supported by results from a study that looked at racial differences in association to insulin sensitivity and oxidative stress in women of African- and European American descent [43]. Thus, we speculate that elevated levels of protein carbonyls in both colorectal adenopolyps and carcinoma from African American patients (**Figure 4**) in this study may play a role in the aggressiveness of the disease in this subgroup displayed in **Table 1**. However, these results do not provide evidence that increased protein carbonyls is solely the cause of differences between African Americans and Caucasians in regards to colorectal tumor aggressiveness. Alterations of the redox imbalance within the cell leading to oxidative damage to proteins, lipids, and nucleic acids cannot be ruled out as a cause of this difference. There were no significant differences were found in the levels of carbonyl proteins between gender samples analyzed as in results. Previous study using animals has found oxidative damage to mitochondrial DNA in males is 4-fold higher than that in females due to higher expression and activities of Mn-superoxide dismutase and of glutathione peroxidase in females, which behave as double transgenics over-expressing superoxide dismutase and glutathione peroxidase, conferring protection against free-radical-mediated damage in aging [44].

To our knowledge, this report is first to show a relationship between early colorectal adenopolyps and elevated reactive protein carbonyls as well as to note differences in expression of reactive protein carbonyls between African American and Caucasian patients bearing the disease. This is noteworthy, since oxidative stress is considered to be triggered by luminal nutrients induced inflammation in the intestine. Inflammation normally leads to production of oxidants to kill pathogens. But these oxidants can also cause damage to DNA, proteins, and lipids and may be, therefore, involved in colorectal tumoriogenesis [24].

Strength of these findings is the similarity in the levels of protein reactive carbonyls of colorectal tumor samples using both Western blot and spectrophotometric measuring techniques. However limitations include the limited population of African Americans with colorectal cancer as well as the small sample size used for each category. Further research including a larger sample size is needed to fully understand the role of oxidative stress on colorectal adenopolyps and racial disparities within the aggressiveness of the disease. In addition to a larger sample size future research should also include a sample of patients of different ethnic backgrounds and focus on the potential involvement of epigenetic regulations such as microRNAs in the regulatory circuitry underlying disparity [45]-[47].

## 5. Conclusion

Results from this study demonstrate an association of elevated levels of reactive protein carbonyls formed by oxidative stress, with colorectal adenopolyps. The results also indicate a racial difference in levels of these carbonyl groups and invasive stages of colorectal carcinoma among patients bearing this disease. Additional research is needed to determine if a higher prevalence of aggressive colorectal carcinomas in African Americans correlates with greater oxidative damage.

## Figures and Tables

**Table 1:** Characteristic of colorectal tumors and patients.

**Characteristics**	Category	Sub-category	N = 41 pairs
Gender	Men		17 (42%)
	Women		21 (51%)
	Unknown		3 (7%)
Ethnicity	African-American		11 (27%)
	Caucasian		27 (66%)
	Unknown		3 (7%)
Age (years)	≥55	African American	9 (28%)
		Caucasian	23 (72%)
		Total	32
	≤55	African American	2 (33%)
		Caucasian	4 (67%)
		Total	6
	Unknown	Total	3
Differentiation	Tubular		7 (12%)
	Tubulovillous		14 (25%)
	Villous		10 (17%)
	Polypvillous		8 (14%)
	Cancer		18 (32%)
